# Comprehensive post-marketing safety evaluation of atezolizumab: A disproportionality analysis based on individual case safety reports in the FAERS

**DOI:** 10.1371/journal.pone.0344190

**Published:** 2026-03-03

**Authors:** Yu Cui, Yuxuan Gao, Na Meng, Xiaojuan Li, Na Zhao, Lili Yu

**Affiliations:** 1 Medical Information Data Department, The 960th Hospital of the PLA Joint Logistics Support Force, Jinan, China; 2 School of Public Health, Shandong Second Medical University, Weifang, China; Keio University School of Medicine Graduate School of Medicine: Keio Gijuku Daigaku Igakubu Daigakuin Igaku Kenkyuka, JAPAN

## Abstract

Atezolizumab is a widely used immune checkpoint inhibitor (ICI) for cancer treatment, and postmarketing testing is important. This study aims to provide a reference for the safe and rational use of drugs in clinical practice by mining and analyzing the adverse event (AE) signals of atezolizumab on the basis of the FDA Adverse Event Reporting System (FAERS). This research extracted AE reports from the second quarter (Q2) of 2016 to Q2 of 2024 from the FAERS. AEs were standardized and classified on the basis of the System Organ Class (SOC) and Preferred Term (PT) from the Medical Dictionary for Regulatory Activities (MedDRA) version 23.0. This study utilized disproportionality analysis (DPA) for signal mining and analysis, including the reporting odds ratio (ROR) method, the Medicines and Healthcare Products Regulatory Agency (MHRA) method, and the Bayesian confidence propagation neural network (BCPNN) method. We obtained a total of 3,124 AE signals and identified 640 PTs and 21 SOCs for atezolizumab. The highest signal intensity was systemic immune activation (n = 15, ROR = 449.20, PRR = 449.07, IC = 8.06), and the most frequently reported AEs were death, pyrexia, infectious pneumonia, anaemia, and febrile neutropenia. The top 100 PTs in terms of signal intensity involved a total of 16 SOCs, including those associated with endocrine disorders; respiratory, thoracic and mediastinal disorders; and renal and urinary disorders. This study revealed that AEs in the endocrine, respiratory and urinary systems need to be monitored in clinical practice.

## Introduction

Atezolizumab is an ICI that has received FDA approval for multiple neoplastic conditions and may be administered as monotherapy or in combination with chemotherapy. Atezolizumab is a fully human immunoglobulin G1 monoclonal antibody that has been successively approved for use in non-small cell lung cancer (NSCLC), small cell lung cancer (SCLC), hepatocellular carcinoma (HCC), melanoma and alveolar soft part sarcoma (ASPS). The FDA approved the combination of atezolizumab with nab-paclitaxel for the treatment of triple-negative breast cancer (TNBC) patients in March 2019, but withdrew this approval in October 2021 after the IMpassion131 trial (NCT03125902) [1] failed to meet its primary end point of progress free survival (PFS) superiority in the frontline treatment of patients with PD-L1 positivity. It binds to programmed death ligand-1 (PD-L1) and blocks the interaction of PD-L1 with programmed cell death protein-1 (PD-1) as well as the B7.1 receptor, relieves the immunosuppression mediated by the PD-1/PD-L1 signaling pathway, and improves the recognition and killing effect of the immune system on tumor cells, thus achieving tumor cell clearance [2]. Although ICIs are widely used and generally demonstrate good patient tolerance and safety, concomitant immune-related adverse effects (irAEs) are gradually becoming a focus of clinical concern, and a comprehensive assessment of the overall safety profile of atezolizumab during the postmarketing surveillance phase is critical.

Atezolizumab has significant clinical efficacy, but its use has also been associated with a number of serious AEs, including death [3] and serious irAEs [4]. A systematic review [5] in 2021 revealed that patients treated with PD-L1 inhibitors developed irAEs at a rate of 74% (14% grade ≥3). A meta-analysis [3] of 112 trials involving 19 217 patients reported toxicity-related fatality rates of 0.38% (PD-L1 inhibitors) and 0.36% (PD-1 inhibitors). A series of premarketing clinical trials of atezolizumab [6,7] reported a portion of common AEs, such as anemia, pyrexia, and hypothyroidism.

Commonly, premarketing clinical trials of drugs have limited sample sizes and short durations and may fail to detect rare or delayed adverse drug events (ADEs). Therefore, it is extremely important to detect and evaluate the actual safety risks of atezolizumab after it is marketed. The FAERS is a self-reported database with an open, informative and relatively reliable source of data, including patient demographics, regression, and medication information. DPA serves to identify signals by comparing the incidence of specific ADEs with background frequencies through statistical methods. This approach has the advantages of processing large amounts of spontaneously reported data, quickly identifying potential safety issues, and being very effective as an initial screening tool despite possible confounding factors. FAERS is real-time and continuous, providing constant data updates to support long-term monitoring. In addition, DPA can run periodically to detect new safety signals in a timely manner. The combination of the two enables continuous assessment of safety after atezolizumab is marketed, filling a gap in premarketing studies.

Therefore, this study summarized the reports of atezolizumab-related AEs on the basis of the FAERS and performed AE signal mining to assess its postmarketing safety issues, with the goal of providing a reference for safe clinical medication.

## Materials and methods

In this study, individual case safety reports (ICSRs) from FAERS, which include the ROR method, the MHRA method, and the BCPNN method, were signal-mined and analyzed via DPA. Since all data of our study was originated from public databases with non-identifable datasets, no ethical approval is required.

### Data source

In this study, ADE reports were extracted from the FAERS for a total of 33 quarters from the second quarter (Q2) of 2016 to Q2 of 2024 in ASCII format. We did not have access to personally identifiable information about the participant during or after data collection.

### Data processing

The data collected in this study included DEMO, DRUG, REAC, OUTC, RPSR, THER, and INDI, totaling 7 types of data and deleted cases. The removal of duplicate reports was performed through the DEMO table according to FDA recommendations [8]; reports with the same CASEID were selected to retain the most recent reports in FDA_DT, and the report with the higher PRIMARYID field was retained when both were the same.

In this study, we standardized the AE reports in the FAERS with reference to the Medical Dictionary for Regulatory Activities (MedDRA), standardized the terminologies for AEs on the basis of PT in the MedDRA, and grouped them on the basis of the SOC.

**Fig 1:**
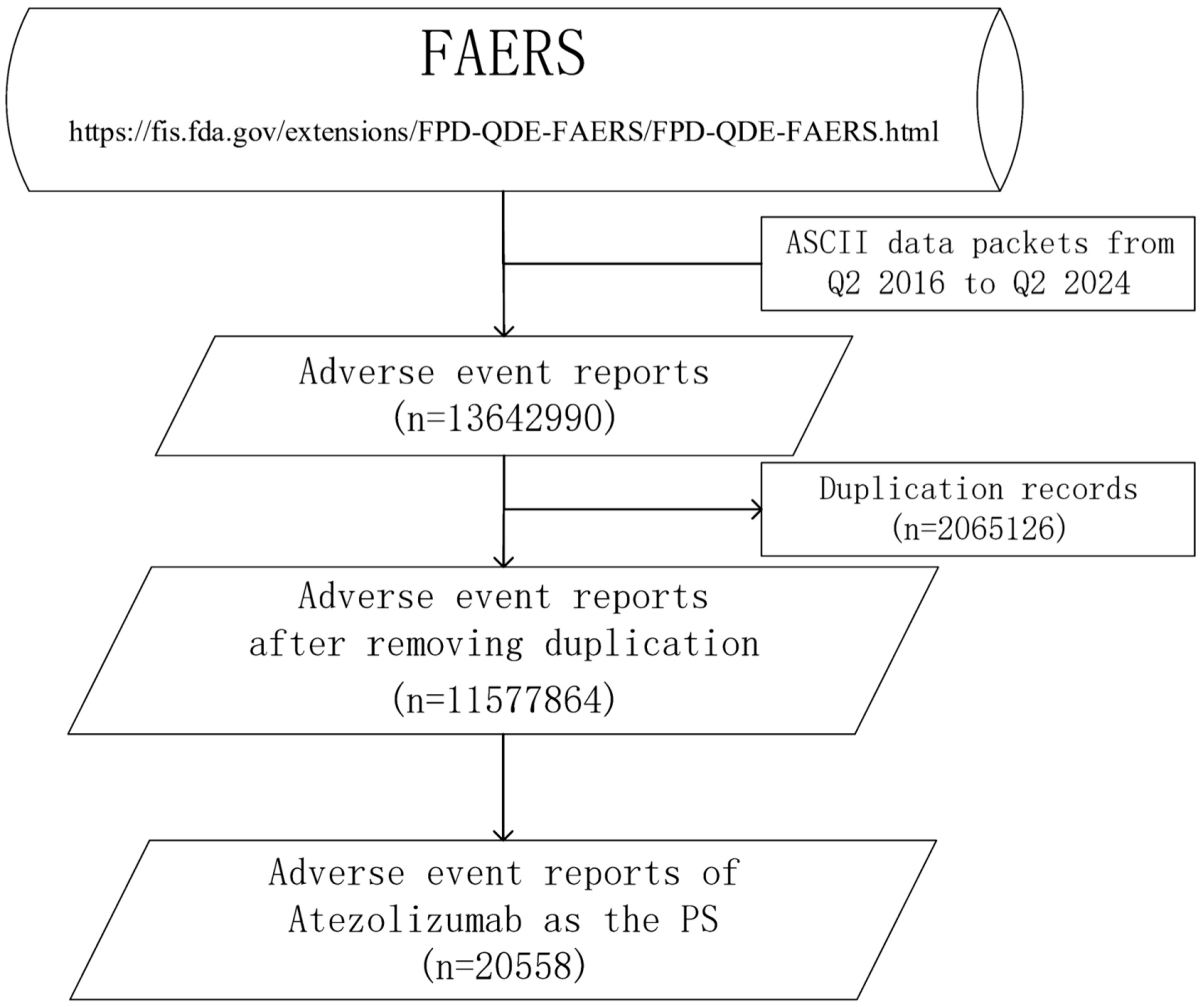
Screening flowchart for atezolizumab AE reports.

The search was conducted with “atezolizumab”, the trade name “Tecentriq”, and the alternative appellation “MPDL3280A”. The ROLE_COD column in the DRUG table provides the level of suspicion for the drug, including “PS” (primary suspicion), “SS” (secondary suspect), “C” (concomitant), and “I” (interactive). To control for confounding factors and minimize the impact of combination therapy, only the reports with the ROLE_COD column were extracted as “PS” (Fig 1).

### Data analysis

DPA is currently the most commonly used class of algorithms in the daily practical work of academic research institutions and drug regulatory authorities in various countries and regions around the world; it has the advantages of simple principles, low algorithmic complexity, and easy implementation [9] and mainly includes the ROR method, the proportional reporting ratio (PRR) method, the MHRA method, and the BCPNN method.

The ROR and PRR methods are frequency counting methods with simple calculation methods and high sensitivity. However, the PRR method is more sensitive and prone to false positives, low specificity and greater bias of results when there are fewer AE reports, and the addition of ꭓ^2^ to the MHRA method can improve the specificity. The BCPNN method is a Bayesian algorithm with high specificity that is capable of performing advanced analyses and can be used to address complex variables, which improves the quantity and quality of drug monitoring; however, it has relatively low sensitivity and suffers from multiple comparisons and biases [10]. The results of one method alone may be highly biased, so it is recommended that multiple algorithms can be used to explore the correlation between drugs and AEs when detecting AE signals [11]. In this study, the ROR, MHRA and BCPNN methods [12] were combined to screen positive signals on the basis of a two-by-two contingency table (Table 1) [13]. The formula and threshold for the MHRA methods are shown in Table 2.

**Table 1:** Two-by-two contingency table for disproportionality analysis.

	Number of target AEs	Number of other AEs	Total
Target drugs	a	b	a + b
Other drugs	c	d	c + d
Total	a + c	b + d	a + b + c + d

**Table 2:** Formulas and thresholds for the MHRA methods.

Method	Formula	Threshold
ROR	$\text{ROR}=\frac{\text{a}/\text{c}}{\text{b}/\text{d}}=\frac{\text{ad}}{\text{bc}}$	① a ≥ 3 ② 95% CI (lower limit)>1
	$\text{95}\%\text{ CI}={\text{e}}^{\text{ln}(\text{ROR})\pm \text{1}.\text{96}\sqrt{\frac{\text{1}}{\text{a}}+\frac{\text{1}}{\text{b}}+\frac{\text{1}}{\text{c}}+\frac{\text{1}}{\text{d}}}}$	
MHRA	$\text{PRR}=\frac{\text{a}/(\text{a}+\text{b})}{\text{c}/(\text{c}+\text{d})}=\frac{\text{a}(\text{c}+\text{d})}{\text{c}(\text{a}+\text{b})}$	① a ≥ 3 ② PRR > 2 ③ $\chi^{\text{2}}$≥4
	$\chi^{\text{2}}=\frac{{(\left|\text{ad}-\text{bc}\right|-\frac{\text{n}}{\text{2}})}^{\text{2}}\text{n}}{(\text{a}+\text{b})(\text{a}+\text{c})(\text{c}+\text{d})(\text{b}+\text{d})}$	
BCPNN	$\text{IC}={\text{log}}_{\text{2}}\frac{\text{p}(\text{y}/\text{x})}{\text{p}(\text{y})}={\text{log}}_{\text{2}}\frac{\text{p}(\text{y},\text{ x})}{\text{p}(\text{x}textrmp(\text{y})}$	① a ≥ 3 ② IC025 > 0
	$\text{E}\left(\text{IC}\right)={\text{log}}_{\text{2}}\frac{\left(\text{a}+\gamma_{\text{11}}\mathrm{\backslash rightleft}(\text{a}+\text{b}+\text{c}+\text{d}+\alpha \right)(\text{a}+\text{b}+\text{c}+\text{d}+\beta )}{(\text{a}+\text{b}+\text{c}+\text{d}+\gamma )(\text{a}+\text{b}+\alpha_{\text{1}})(\text{a}+\text{c}+\beta_{\text{1}})}$	
	$\text{V}\left(\text{IC}\right)=\frac{\text{1}}{{\left(\text{ln2}\right)}^{\text{2}}}\left[\frac{\text{b}+\text{c}+\text{d}+\gamma -\gamma_{\text{11}}}{\left(\text{a}+\gamma_{\text{11}}\mathrm{\backslash rightleft}(\text{1}+\text{a}+\text{b}+\text{c}+\text{d}+\gamma \right)}+\frac{\text{c}+\text{d}+\alpha -\alpha_{\text{1}}}{\left(\text{a}+\text{b}+\alpha_{\text{1}}\right)\left(\text{1}+\text{a}+\text{b}+\text{c}+\text{d}+\alpha \right)}+\frac{\text{b}+\text{d}+\beta -\beta_{\text{1}}}{\left(\text{a}+\text{c}+\beta_{\text{1}}\right)\left(\text{1}+\text{a}+\text{b}+\text{c}+\text{d}+\beta \right)}\right]$	
	$\gamma =\gamma_{\text{11}}\frac{(\text{a}+\text{b}+\text{c}+\text{d}+\alpha )(\text{a}+\text{b}+\text{c}+\text{d}+\beta )}{(\text{a}+\text{b}+\alpha_{\text{1}})(\text{a}+\text{c}+\beta_{\text{1}})}$	
	$\text{IC025}=\text{IC}-\text{2SD}=\text{E}\left(\text{IC}\right)-\text{2}\sqrt{\text{V}(\text{IC})}$	

## Results

### Descriptive analysis of the AE reports

**Fig 2:**
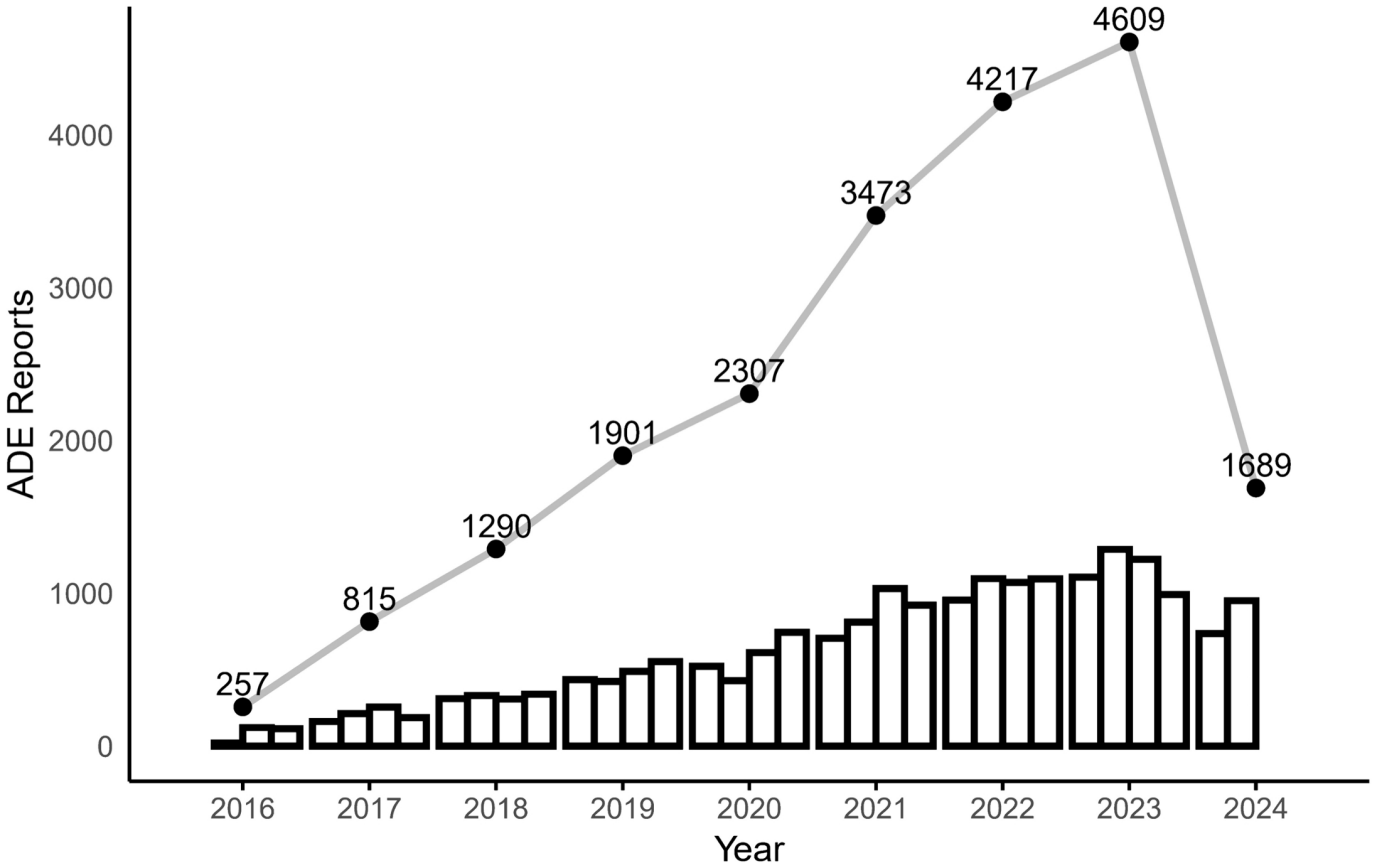
Trends in the number of AE reports for atezolizumab (2016 Q2--2024 Q2).

**Fig 3:**
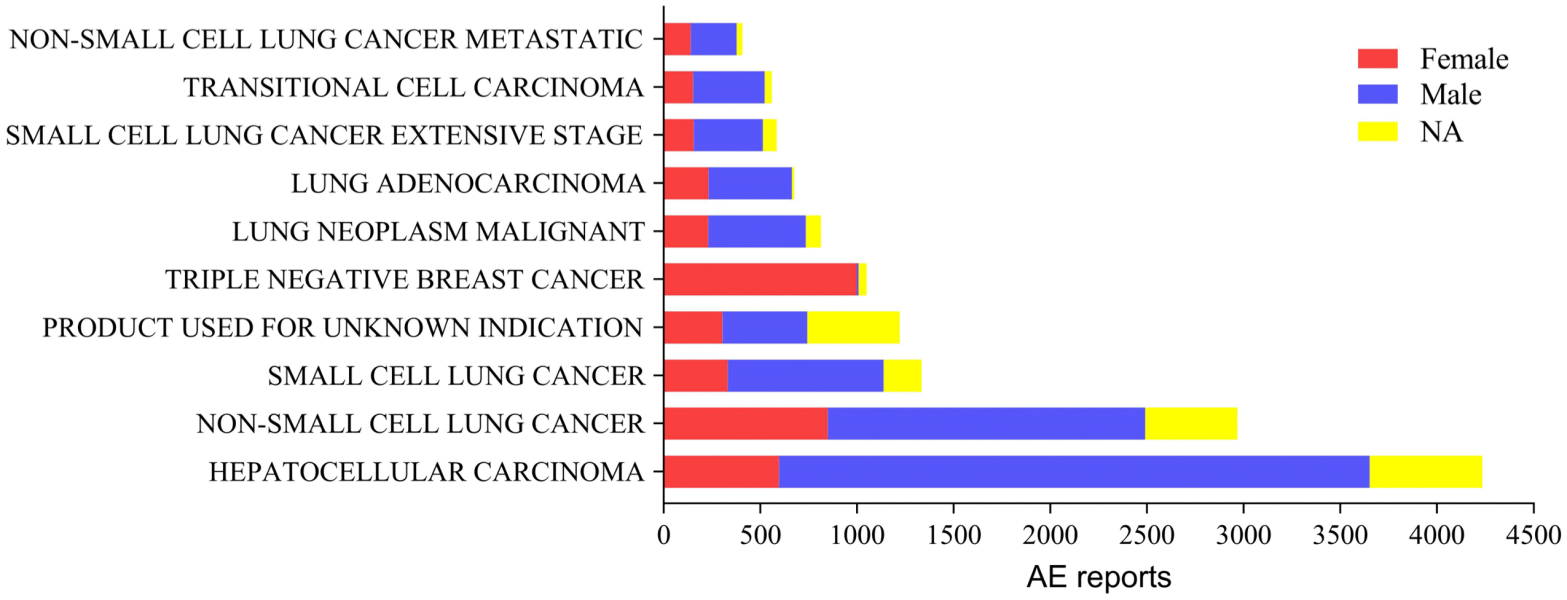
Gender distribution of AE reporting indications.

In this study, 13,642,990 ADE reports were extracted from the FAERS; after deduplication, the final result was 1,157,7864 reports, and 20,558 AE reports were retrieved, for which atezolizumab was the first suspected drug. The number of AE reports after the marketing of atezolizumab has shown a relatively rapid increase, with the largest increase occurring in 2021 (Fig 2). The highest number of reports were from Japan, followed by the United States. There were more male than female patients in the reports, the age group was mainly above 65 years, the reporters were mainly physicians, 22.64% of the ADEs reported a treatment outcome of death, and bevacizumab is the drug with the most concomitant use of atezolizumab (Table 3). We describe the sex distribution of the most reported drug indications, with more male than female patients among all but triple-negative breast cancer patients (Fig 3).

**Table 3:** Demographic information of atezolizumab AE reports.

Factors	Number (%)
**Gender**	
Female	6946 (33.79)
Male	10965 (53.34)
Unknown	2647 (12.88)
**Age**	
<18	167 (0.81)
18~<65	6279 (30.54)
65~≤85	7721 (37.56)
>85	192 (0.93)
Unknown	6199 (30.15)
**Serious Outcome**	
Death	4655 (22.64)
Hospitalized	6909 (33.61)
Other Serious Outcome	6675 (32.47)
Unknown	2319 (11.28)
**Concomitant Medications**	
Bevacizumab	7854
Carboplatin	5154
Paclitaxel	3902
Acetaminophen	2423
Etoposide	2041
Dexamethasone	1614
Amlodipine Besylate	1051
Ondansetron	1017
Furosemide	1000
Omeprazole	940
**Reported Countries**	
Japan	5644 (27.45)
United States	4370 (21.26)
France	1174 (5.71)
India	1028 (5.00)
China	1023 (4.98)
Others	7319 (35.60)
**Reported Person**	
Physician	15541 (75.60)
Consumer	2076 (10.10)
Health Professional	1595 (7.76)
Pharmacist	965 (4.69)
Others	317 (1.54)
Unknown	64 (0.31)
**Indications**	
Hepatocellular Carcinoma	4234
Non-Small Cell Lung Cancer	2968
Small Cell Lung Cancer	1334
Product Used for Unknown Indication	1221
Triple Negative Breast Cancer	1050
Lung Neoplasm Malignant	814
Lung Adenocarcinoma	676
Small Cell Lung Cancer Extensive Stage	585
Transitional Cell Carcinoma	559
Non-Small Cell Lung Cancer Metastatic	408

### Analysis of the AE signals

There were 3124 PT signals involved in the reporting of atezolizumab as a PS, which were then screened via the above ROR, MHRA and BCPNN methods. Finally, 640 PTs were obtained after the signals related to product quality, use problems and drug indications were removed. We performed a parallel data mining analysis of atezolizumab using the Japanese Adverse Drug Event Report (JADER), which is independent of the FAERS. We applied the same signal detection method and inclusion/exclusion criteria as those used in the FAERS analysis to ensure methodological consistency. A total of 1092 PT signals involved in the reporting of atezolizumab in the JADER, and 89 PTs were obtained in JADER, with 78 PTs (87.64%) overlapping those in FAERS (Table 4 and 5).

**Table 4:** The 20 most frequently reported PTs detected by both FAERS and JADER.

PT	FAERS				JADER			
	n	ROR(95%Cl)	PRR($\chi^{2}$)	IC(IC025)	n	ROR(95%Cl)	PRR($\chi^{2}$)	IC(IC025)
Febrile Neutropenia	577	10.58(9.74-11.49)	10.47(4871.83)	3.37(3.25)	422	3.45(3.12-3.82)	3.32(662.45)	1.68(0.02)
Pneumonitis	527	22.82(20.91-24.90)	22.59(10521.02)	4.45(4.32)	167	3.70(3.16-4.33)	3.64(304.63)	1.81(0.14)
Colitis	398	12.67(11.47-14.00)	12.58(4166.44)	3.63(3.48)	106	4.95(4.05-6.03)	4.89(305.96)	2.21(0.54)
Hypertension	385	2.37(2.15-2.62)	2.36(302.34)	1.24(1.09)	260	6.35(5.58-7.23)	6.18(1035.40)	2.52(0.85)
Adrenal Insufficiency	306	30.55(27.24-34.28)	30.38(8311.52)	4.86(4.69)	251	4.28(3.76-4.87)	4.18(574.31)	1.99(0.33)
Proteinuria	291	19.07(16.96-21.43)	18.96(4814.61)	4.21(4.03)	351	21.40(18.93-24.18)	20.51(4943.62)	3.98(2.31)
Ascites	284	12.96(11.52-14.58)	12.89(3057.19)	3.66(3.49)	130	6.67(5.56-8.00)	6.58(558.98)	2.60(0.93)
Hepatitis	243	13.25(11.67-15.05)	13.20(2686.13)	3.70(3.51)	36	4.93(3.51-6.92)	4.91(104.26)	2.21(0.54)
Hepatic Failure	189	9.38(8.13-10.83)	9.35(1390.40)	3.21(3.00)	74	6.24(4.91-7.93)	6.19(294.24)	2.52(0.85)
Encephalitis	162	30.14(25.74-35.29)	30.05(4351.11)	4.85(4.62)	119	15.95(13.04-19.52)	15.73(1318.8)	3.68(2.01)
Myositis	149	24.03(20.40-28.30)	23.96(3163.48)	4.53(4.29)	69	8.86(6.88-11.41)	8.79(419.45)	2.97(1.30)
Cholangitis	111	23.71(19.62-28.66)	23.66(2325.61)	4.52(4.24)	59	5.24(4.02-6.84)	5.21(185.91)	2.29(0.62)
Nephritis	107	39.28(32.31-47.74)	39.20(3759.20)	5.21(4.93)	10	4.15(2.19-7.87)	4.15(22.43)	1.98(0.30)
Type 1 Diabetes Mellitus	105	23.78(19.57-28.90)	23.73(2207.03)	4.52(4.23)	105	5.65(4.62-6.90)	5.59(364.58)	2.38(0.72)
Hepatic Encephalopathy	105	15.25(12.56-18.50)	15.22(1363.28)	3.9(3.61)	57	5.42(4.14-7.11)	5.39(188.33)	2.34(0.67)
Skin Disorder	102	3.56(2.93-4.33)	3.56(186.59)	1.83(1.54)	61	6.29(4.83-8.19)	6.25(245.48)	2.53(0.86)
Myasthenia Gravis	86	13.15(10.62-16.28)	13.13(944.77)	3.69(3.38)	46	3.87(2.87-5.22)	3.85(91.87)	1.88(0.22)
Peripheral Sensory Neuropathy	75	16.48(13.10-20.72)	16.45(1062.24)	4.01(3.67)	23	9.15(5.91-14.16)	9.12(145.60)	3.02(1.34)
Haemophagocytic Lymphohistiocytosis	73	10.19(8.09-12.84)	10.18(595.05)	3.33(2.99)	81	5.76(4.58-7.23)	5.71(289.36)	2.41(0.74)
Oesophageal Varices Haemorrhage	69	44.7(35.02-57.05)	44.64(2757.09)	5.39(5.03)	89	32.09(24.87-41.42)	31.75(1771.30)	4.43(2.75)

**Table 5:** PTs determined to be positive signals only in JADER.

PT	FAERS					JADER		
	n	ROR(95%Cl)	PRR($\chi^{2}$)	IC(IC025)	n	ROR(95%Cl)	PRR($\chi^{2}$)	IC(IC025)
Hepatocellular Injury	28	1.88(1.30-2.73)	1.88(11.51)	0.91(0.37)	6	9.59(4.06-22.61)	9.58(40.09)	3.08(1.36)
Influenza Like Illness	59	1.05(0.81-1.35)	1.05(0.13)	0.07(−0.31)	5	11.02(4.26-28.47)	11.01(38.82)	3.25(1.52)
Gingivitis	6	1.52(0.68-3.39)	1.52(1.06)	0.60(−0.49)	4	3.81(1.39-10.46)	3.81(7.83)	1.87(0.17)
Subdural Hygroma	2	8.18(2.03-33.00)	8.18(12.45)	3.02(1.34)	4	17.04(5.65-51.34)	17.03(47.65)	3.77(1.98)
Glossitis	4	1.59(0.60-4.25)	1.59(0.89)	0.67(−0.62)	3	4.91(1.52-15.90)	4.91(8.68)	2.21(0.49)
Henoch-Schonlein Purpura Nephritis	2	24.86(6.06-102.00)	24.85(44.12)	4.58(2.87)	3	8.71(2.61-29.11)	8.71(18.01)	2.96(1.20)
Hepatic Infarction	2	7.16(1.78-28.84)	7.16(10.48)	2.83(1.15)	3	7.37(2.23-24.36)	7.37(14.80)	2.75(1.00)
Gastroduodenal Ulcer	2	6.46(1.60-26.00)	6.46(9.13)	2.68(1.00)	3	5.04(1.56-16.34)	5.04(9.01)	2.25(0.52)
Meningitis Noninfective	1	19.37(2.65-141.52)	19.37(16.93)	4.24(2.14)	3	21.29(5.76-78.67)	21.29(43.50)	4.02(2.17)
Glomerulonephritis Acute	1	6.59(0.92-47.22)	6.59(4.69)	2.71(0.65)	3	9.58(2.85-32.25)	9.58(20.04)	3.08(1.31)
Anorectal Varices Haemorrhage	–	–	–	–	3	191.65(19.93-1842.68)	191.58(142.19)	5.60(3.50)

**Fig 4:**
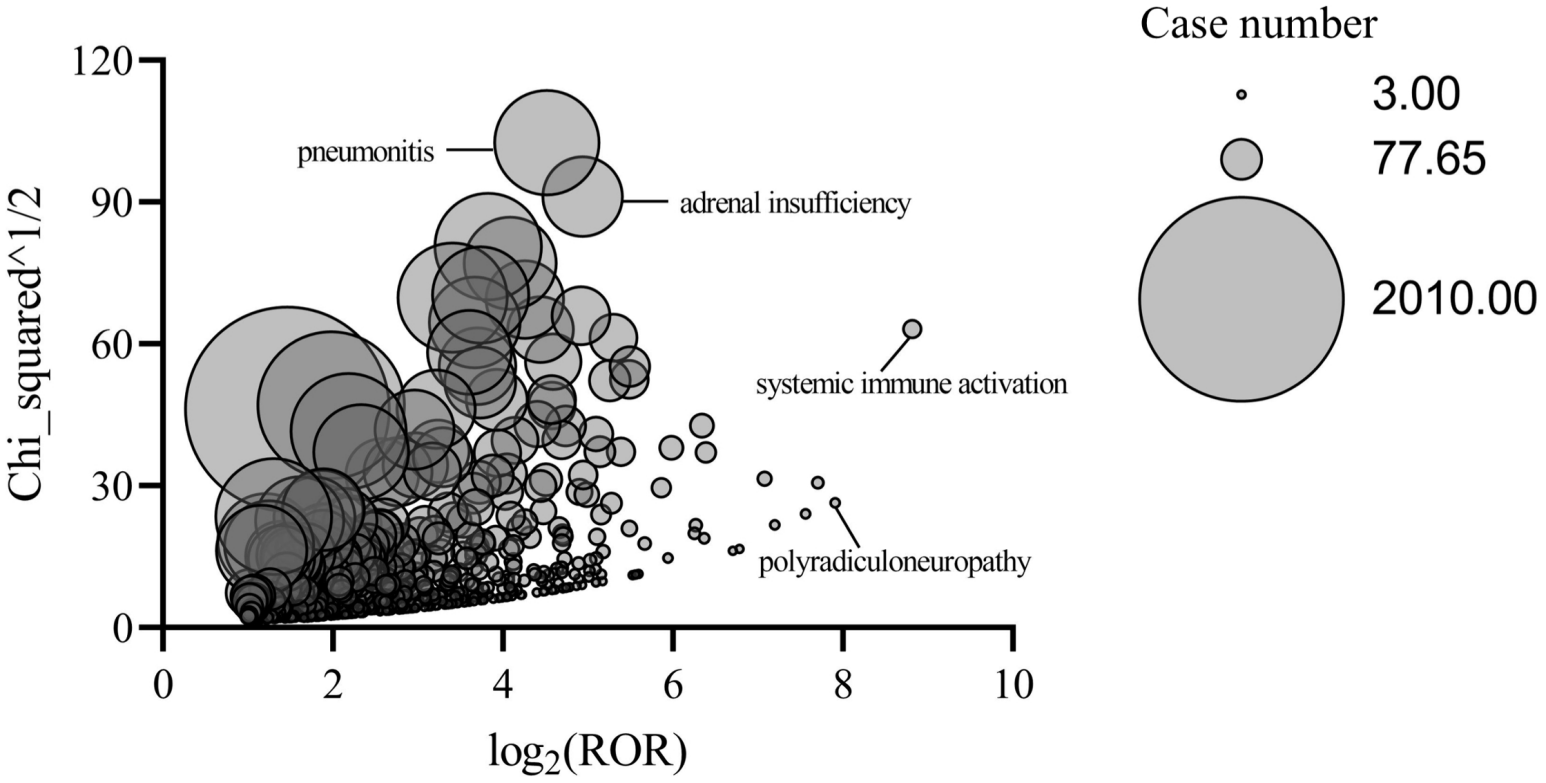
Distribution of AE signals in response to atezolizumab.

**Fig 5:**
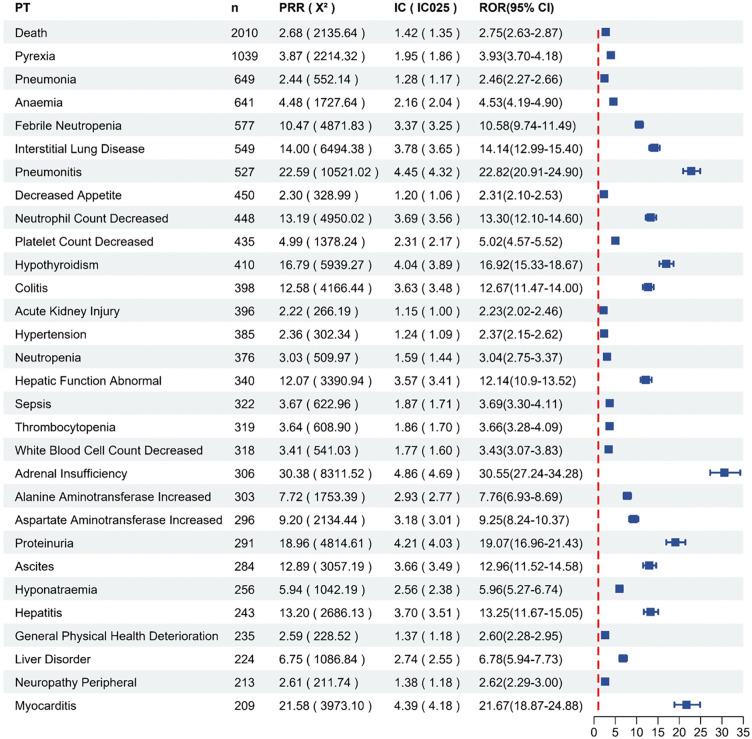
Top 30 AEs with the highest percentage of signal detection in the FAERS (PT = Preferred Term, PRR = Proportional Reporting Ratio, ROR = Reporting Odds Ratio, IC = Information Component).

The distribution of AE signals in response to atezolizumab is shown in Fig 4. The most intense AE signal was systemic immune activation (n = 15, ROR = 449.20, PRR = 449.07, IC = 8.06). We focused on the top 30 most frequently occurring AE signals (Fig 5). The 5 most frequently reported AEs for atezolizumab were death, fever pyrexia, pneumonia, anemia, and febrile neutropenia. It is worth noting that death is a commonly reported outcome, but in self-reported databases, it typically encompasses events related to disease progression and does not necessarily indicate a direct cause by the drug.

### AE reports stratified by SOC

**Fig 6:**
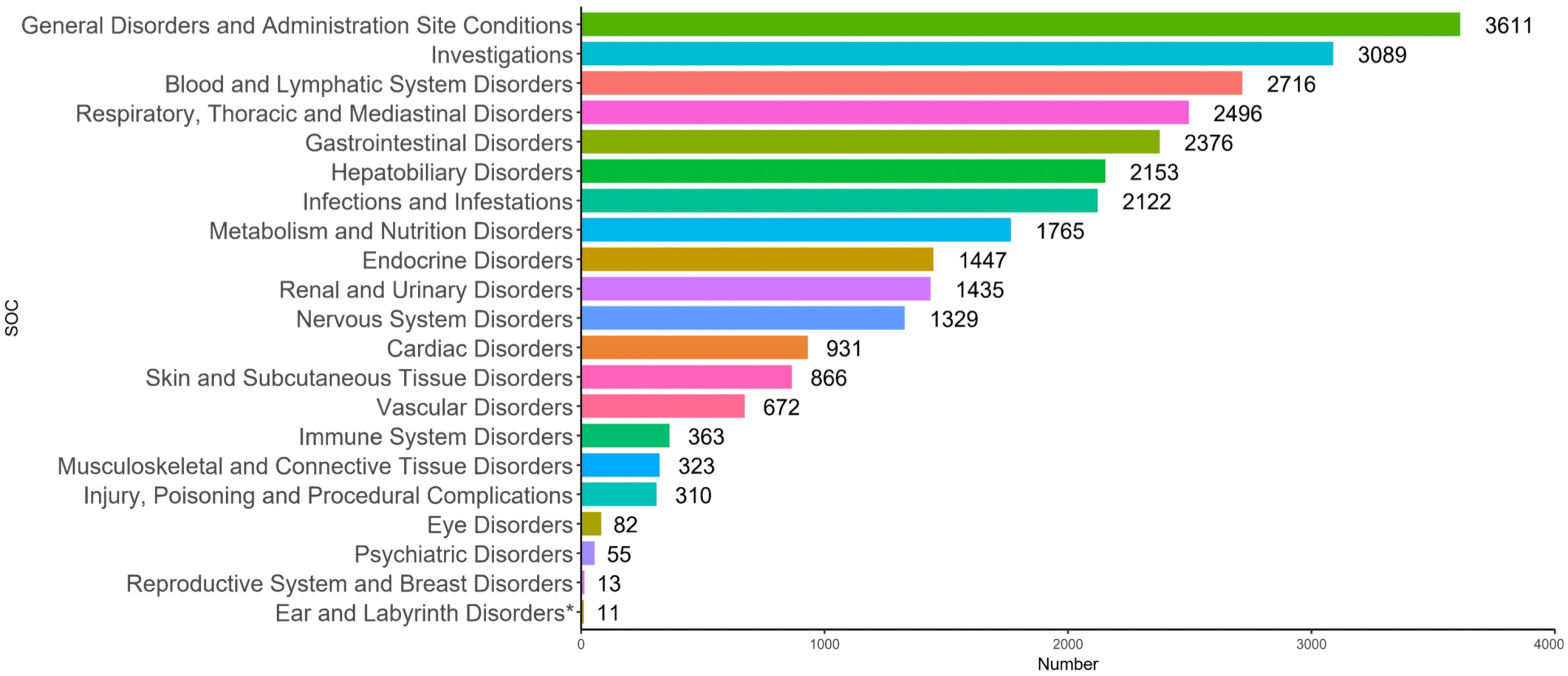
Atezolizumab AE reports involving SOCs. * Indicates SOC not mentioned in the drug instructions.

The 640 PTs screened for atezolizumab-related AE reports involved 21 SOCs. The SOCs with the highest cumulative number of reports included general disorders and administration site conditions (12.82%), investigations (10.97%), blood and lymphatic system disorders (9.64%), and blood and lymphatic system disorders (9.64%) (Fig 6).

**Fig 7:**
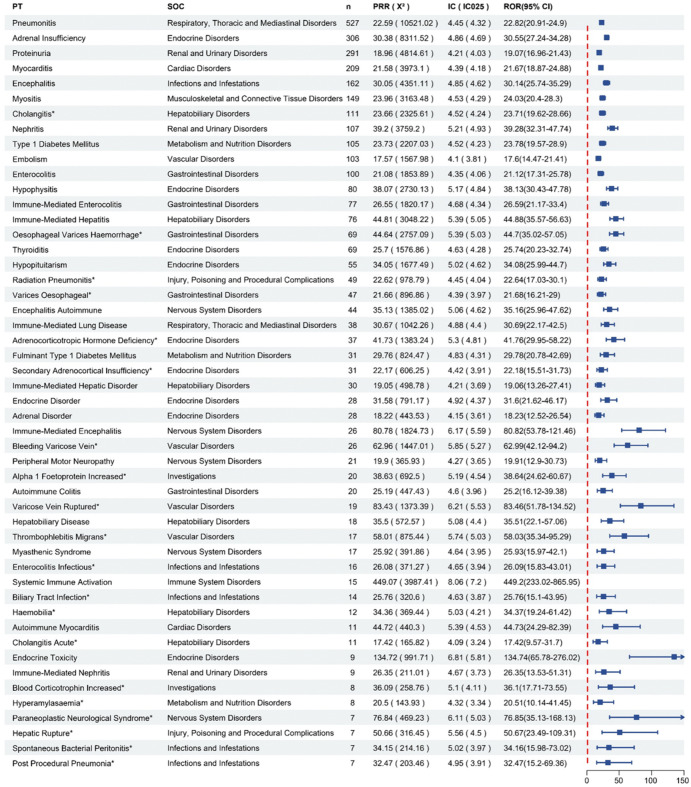
Top 50 AEs in terms of IC (PT = Preferred Term, SOC = System Organ Class, PRR = Proportional Reporting Ratio, ROR = Reporting Odds Ratio, IC = Information Component). * Indicates PT not mentioned in the drug instructions.

Focusing on the 15 SOCs involved in the top 50 PTs in terms of IC (Fig 7). A comparison with the prescribing information for atezolizumab revealed that varices oesophageal (n = 47, ROR = 21.68, PRR = 21.66, IC = 4.39) and cholangitis (n = 111, ROR = 23.71, PRR = 23.66, IC = 4.52) were not mentioned in the prescribing information and may be potential AEs.

**Fig 8:**
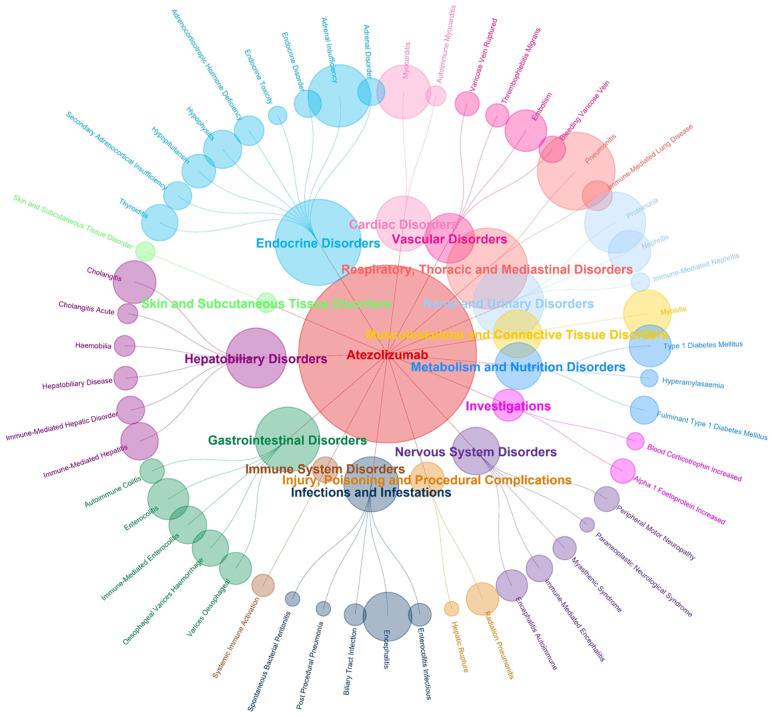
SOC distribution network plot of the top 50 atezolizumab AEs.

**Fig 9:**
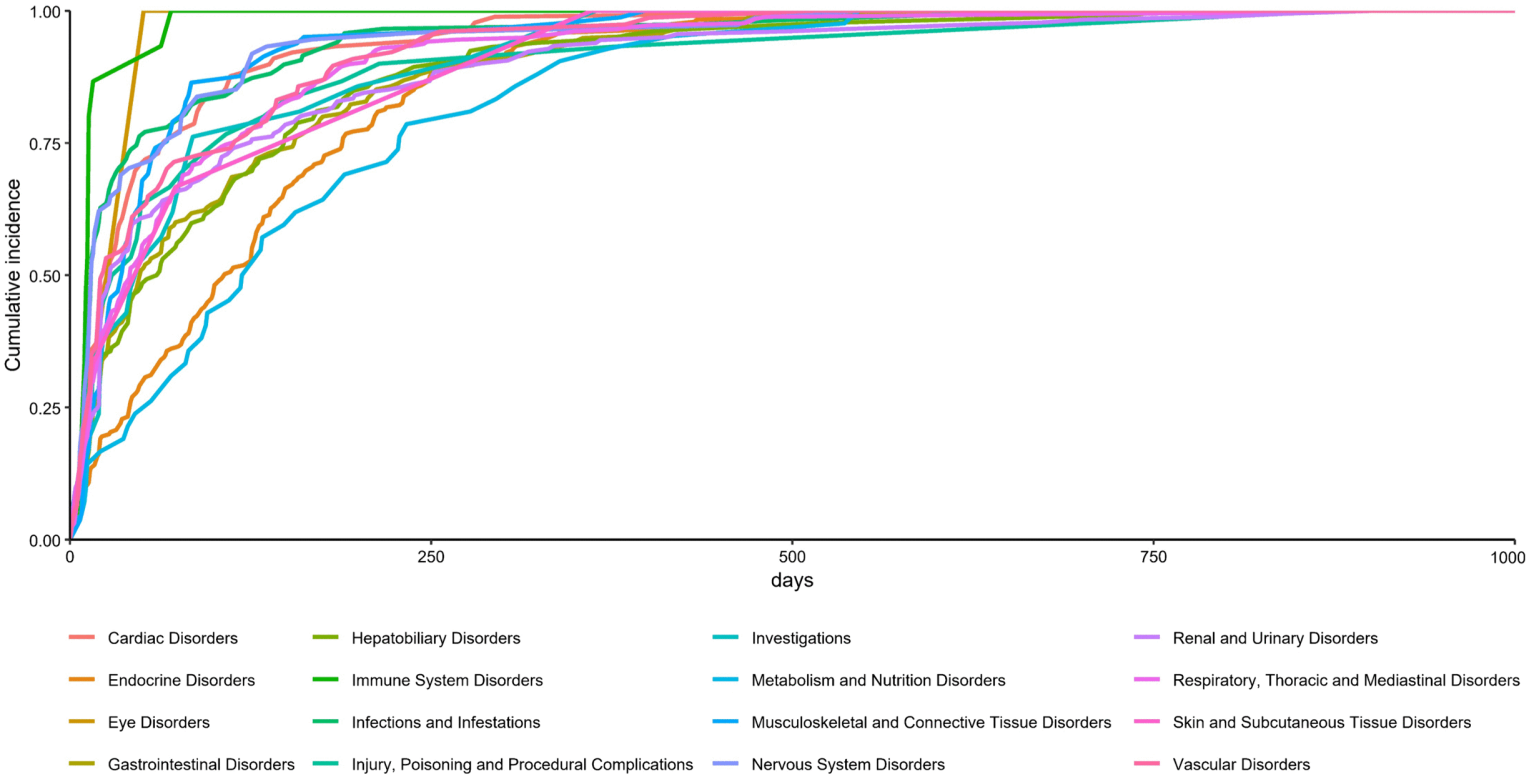
SOC cumulative occurrence time of the top 100 atezolizumab AEs.

The highest number of reports and types of PTs involved were related to endocrine disorders. In addition, respiratory, thoracic and mediastinal disorders and renal and urinary disorders were reported in greater numbers. Nervous system disorders, gastrointestinal disorders and hepatobiliary disorders involve more types of PTs (Fig 8). Metabolism and nutrition disorders and endocrine system disorders are associated with later AEs, whereas immune system disorders and eye diseases occur earlier (Fig 9).

## Discussion

Unlike other studies of atezolizumab based on FAERS [14,15], this study was an assessment of the overall safety of atezolizumab rather than a selection of indications or SOCs for the occurrence of AEs. Therefore, this study was able to analyze the postmarketing safety of atezolizumab more comprehensively.

Atezolizumab is the first PD-L1 inhibitor approved for marketing worldwide, and the number of reported AEs associated with it has increased relatively rapidly since its launch in May 2016. The largest increase in 2021 was likely due to the drug’s approval by the FDA in May 2020 for an expanded indication: first-line treatment of metastatic NSCLC with high PD-L1 expression [16]. The reported indications are predominantly HCC and lung cancer, and studies have shown that the incidence rates of HCC are at least 2 or 3 times higher in males than in females [17], and the incidence of lung cancer is also much higher in men [18]. Therefore, the greater proportion of male patients in atezolizumab AE reports may be partly explained by sex-based differences in the incidence of its primary indications, HCC and lung cancer.

To verify the generalizability of the signals identified in FAERS, we replicated the same data mining protocol in the independent JADER database. A total of 89 PT signals were detected in JADER, with 78 PTs (87.64%) overlapping those in FAERS. This high consistency confirms that the core AEs of atezolizumab exhibit stability across different regions and populations. However, significant differences in signal strength were observed between the two databases. This discrepancy can be attributed to three key factors: (1) Ethnic and population differences: Genetic and immunological variations between Western and East Asian populations may lead to differential susceptibility to adverse events, affecting reporting frequency [19]. (2) Database reporting biases: Variations in adverse event reporting practices and database size between FAERS and JADER may influence signal strength metrics. For example, hepatocellular injury was reported in 28 cases in FAERS and 6 cases in JADER, but it was identified as a positive signal by the ROR method, MHRA method, and BCPNN method simultaneously only in JADER. In FAERS, it was not identified as a positive signal by the MHRA method. (3) Regional differences in clinical practice: Disparities in approved indications, treatment regimens, and concomitant medication use of atezolizumab may alter the profile of reported adverse events. For example, the U.S. FDA approved atezolizumab in combination with cobimetinib and vemurafenib for the treatment of adult patients with BRAF V600 mutation-positive unresectable or metastatic melanoma in 2020 [20], but this indication has yet to receive approval from Japan’s Pharmaceuticals and Medical Devices Agency (PMDA).

During the course of the study, the most frequent AE reports screened were death, pyrexia, infectious pneumonia, anaemia, and febrile neutropenia, with both pyrexia [2] and anaemia [16] as the most common AEs of atezolizumab. A Japanese study suggested that atezolizumab-containing therapies may increase the risk of febrile neutropenia [21]. A pivotal phase III, multicenter, open-label, randomized clinical trial, which demonstrated the efficacy and safety of the treatment of chemotherapy combined with atezolizumab [22], demonstrated that febrile neutropenia is a common grade 3 or 4 treatment-related AE, with a less than 10% higher incidence with the addition of atezolizumab to the treatment. Deaths have been reported in high numbers in this study, but their occurrence may reflect progression of underlying disease in patients with advanced tumors rather than being entirely attributable to atezolizumab. Future studies should utilize clinical records to distinguish treatment-related deaths from disease-related deaths.

Atezolizumab AEs involve multiple organs and systems in the real world, and this study focused on the systems involved in AEs with higher ICs. The total number and types of AEs reported for endocrine disorders were high, occurring mainly in the adrenal gland, hypophysis, and thyroid, which is consistent with its premarketing clinical trials and reports in the prescribing information. Our study revealed that, unlike other ICIs, atezolizumab AEs in the endocrine system occurred most frequently in the adrenal gland, whereas other ICIs affected the thyroid and pituitary glands the most [23]. Clinical attention should be given to monitoring cortisol levels in patients, and because primary adrenal insufficiency can have serious and even life-threatening consequences, adrenocorticotropic hormones should be monitored to distinguish it from secondary adrenocortical insufficiency [24].

AEs occurring in the respiratory system are also of concern, especially pneumonia. Its occurrence is closely related to the patient’s own type of cancer and is frequently common in NSCLC and SCLC, which is the most common serious AE or even leads to the cessation of medication [25]. The prescribing information for atezolizumab reported that patients with prior chest radiotherapy had a higher pneumonia incidence, and studies [26] have provided the same report. This may be due to the damage to lung function caused by radiation therapy at certain doses, the sustained low-level release of inflammatory factors induced by radiation therapy, and the elevated level of inflammatory factors promoted by ICIs [27]. Among other ICIs, the most common SOCs for cemiplimab are respiratory, thoracic and mediastinal disorders, accounting for 11.6% of all SOCs [28]. Immune-related pneumonitis is a rare but fatally threatening serious AE that accounts for 35% of PD-1/PD-L1 inhibitor-related deaths [3]. The mainstay of therapy remains glucocorticoids, which should be followed by a slow taper that requires more than 4 weeks (sometimes 6–8 weeks or longer) to prevent the recurrence of irAEs [29].

Proteinuria and nephritis are the main AEs in renal and urinary disorders. Proteinuria is a common AE in systemic therapy for HCC, and in 2020, the FDA approved atezolizumab plus bevacizumab for the treatment of patients with advanced unresectable or metastatic HCC who had not received prior systemic therapy [30], which was also the first FDA-approved immunotherapy for the first-line treatment of HCC. IMbrave150 [31] reported a 20% incidence of all grades of proteinuria and a 3% incidence of grade ≥3 proteinuria for atezolizumab plus bevacizumab. However, one study [32] reported that proteinuria before treatment with this therapy was an adverse prognostic factor that was independent of liver function and HCC progression. A meta-analysis [33] reported that for nephritis, atezolizumab was the worst-ranked single ICI, with an estimated absolute event rate of 267 cases of severe nephritis per 10,000 patients (95% CI 11--7532). The incidence of ICI-related acute kidney injury (AKI) is estimated to be 2.2–3.5% in individuals treated with PD-1 inhibitors [34,35] and increases significantly to approximately 5% when combination therapy is used [36]. Treatment of irAKI consists of discontinuing the drug in question, as well as administering a course of systemic steroids, with full recovery in the majority of cases, especially when the treatment is initiated early (<3 days after irAKI) [37].

Additionally, varices oesophageal, the more commonly reported AE in gastrointestinal disorders, and cholangitis, the most commonly reported AE in hepatobiliary disorders, are noteworthy. Both of them have been reported to be associated with a variety of PD-1/PD-L1 inhibitor AEs but are not included in the atezolizumab prescribing information. Varices oesophageal has been associated with some case reports [38], occurring in patients with HCC treated with atezolizumab plus bevacizumab. Atezolizumab may stimulate innate immune cells and increase the production of proinflammatory cytokines, which further promotes liver fibrosis, leading to the occurrence of varices oesophageal [39]. Currently, case reports of ICI-associated cholangitis are mostly due to PD-1 inhibitors, while atezolizumab, as a PD-L1 inhibitor, has been reported less frequently [40], which generally occurs late, mostly after the 5th course of ICI or even after termination of treatment [41], so long-term monitoring by clinicians is needed. Large-duct cholangitis induced by ICIs is a very rare condition while small-duct cholangitis is more common, and accounts for a significant number of cases previously diagnosed as “hepatitis” [42]. ICIs-induced cholangitis may exhibit bile duct dilatation or obstructive changes on imaging, along with portal-based inflammation and bile-duct injury [43]. Additionally, predominant CD8 + T cell infiltration is a characteristic feature of ICI-induced cholangitis [44].

The data used in this study are extensive and cover a wide range of populations and are based on real-world data; however, several limitations remain. First, it cannot establish causality; second, adverse reactions may be underreported, particularly mild or common events; third, concomitant medications and underlying conditions may confound signals. Additionally, the FAERS and JADER are self-reporting systems that have incomplete and unstandardized reports and do not allow for the calculation of the incidence of AEs [45]. Nonetheless, the results of this comprehensive signal mining study provide guidance for the safe and rational use of medications. All stakeholders should recognize that DPA is only one step in identifying safety signals, that the signals found are hypotheses rather than proven signals, and that the results do not directly prove causality but need to be combined with clinical reviews and other evidence to validate the signals [46].

## Conclusion

In this study, the ROR, MHRA and BCPNN methods were combined for signal mining analysis of atezolizumab AE reports in the FAERS. We found that it involves a range of organs and systems, especially the endocrine, respiratory and urinary systems, which can provide a reference for the safety of atezolizumab in the clinic.
